# Protective Effect of Tempol against Cisplatin-Induced Ototoxicity

**DOI:** 10.3390/ijms17111931

**Published:** 2016-11-18

**Authors:** Cha Kyung Youn, Jun Kim, Eu-Ri Jo, Jeonghyun Oh, Nam Yong Do, Sung Il Cho

**Affiliations:** 1 Department of Otolaryngology-Head and Neck Surgery, Chosun University School of Medicine, Gwangju 61453, Korea; threefold@hanmail.net (C.K.Y.); mdjunek@medimail.co.kr (J.K.); maria2046@hanmail.net (E.-R.J.); way2ojay@naver.com (J.O.); nydo@chosun.ac.kr (N.Y.D.); 2 Division of Natural Medical Science, Chosun University School of Medicine, Gwangju 61452, Korea

**Keywords:** cisplatin, ototoxicity, cell death, Tempol, apoptosis

## Abstract

One of the major adverse effects of cisplatin chemotherapy is hearing loss. Cisplatin-induced ototoxicity hampers treatment because it often necessitates dose reduction, which decreases cisplatin efficacy. This study was performed to investigate the effect of Tempol on cisplatin-induced ototoxicity in an auditory cell line, House Ear Institute-Organ of Corti 1 (HEI-OC1). Cultured HEI-OC1 cells were exposed to 30 μM cisplatin for 24 h with or without a 2 h pre-treatment with Tempol. Cell viability was determined using 3-[4,5-dimethylthiazol-2-yl]-2,5-diphenyltetrazolium bromide (MTT) assay and apoptotic cells were identified using terminal deoxynucleotidyl transferase dUTP nick end labeling of nuclei (TUNEL) assay and flow cytometry. The effects of Tempol on cisplatin-induced cleaved poly(ADP-ribose) polymerase, cleaved caspase, and mitochondrial inducible nitric oxide synthase expression were evaluated using western blot analysis. Levels of intracellular reactive oxygen species (ROS) were measured to assess the effects of Tempol on cisplatin-induced ROS accumulation. Mitochondria were evaluated by confocal microscopy, and the mitochondrial membrane potential was measured to investigate whether Tempol protected against cisplatin-induced mitochondrial dysfunction. Cisplatin treatment decreased cell viability, and increased apoptotic features and markers, ROS accumulation, and mitochondrial dysfunction. Tempol pre-treatment before cisplatin exposure significantly inhibited all these cisplatin-induced effects. These results demonstrate that Tempol inhibits cisplatin-induced cytotoxicity in HEI-OC1, and could play a preventive role against cisplatin-induced ototoxicity.

## 1. Introduction

Cisplatin is effective for the treatment of solid tumors in the ovaries, testes, lungs, and bladder, as well as for head and neck cancers. However, despite its excellent efficacy, cisplatin use is limited because of the hearing loss caused by its ototoxic effects [1], which necessitates dose reduction and consequently reduces its efficacy as an anticancer agent. Although the mechanism of cisplatin-induced ototoxicity has not yet been completely elucidated, it can mostly be attributed to an increase in the level of reactive oxygen species (ROS), which causes a deficiency of intracochlear antioxidants [2] and induces calcium inflow into hair cells, resulting in apoptosis [3].

Tempol (4-hydroxy-2,2,6,6-tetramethylpiperidinyl-1-oxyl), a nitroxide compound, functions as a transmembranous radical scavenger, similar to superoxide dismutase (SOD) or catalase. Tempol has been reported to restore the intracellular oxidative balance and reduce oxidative stress, resulting in alleviation of nephrotoxicity in mice [4], and reduced infarct size in rat and rabbit myocardial infarction models [5]. Moreover, it exerts a protective effect against lymphocyte genotoxicity [6]. Furthermore, Tempol can easily penetrate the blood–brain barrier and exert neuroprotective effects [7]. Since Tempol acts as an antioxidant, it may be able to suppress cisplatin-induced oxidative stress.

Therefore, in this study, we aimed to ascertain whether Tempol could inhibit ROS-induced apoptosis, which may be the mechanism of cisplatin-induced ototoxicity, and to examine the potential use of Tempol as a protective agent against cisplatin-induced hearing loss.

## 2. Results

### 2.1. Effect of Tempol on the Viability of House Ear Institute-Organ of Corti 1 (HEI-OC1) Cells

**Figure 1:**
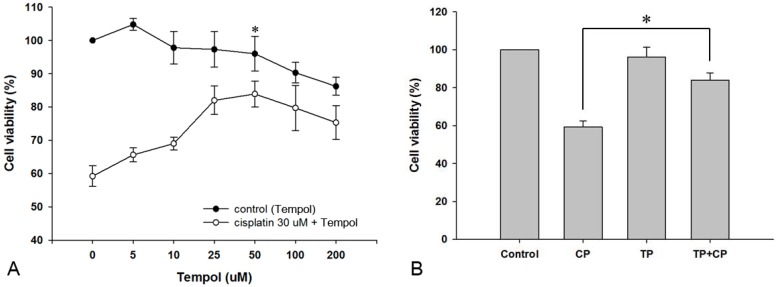
Effect of Tempol on the viability of House Ear Institute-Organ of Corti 1 (HEI-OC1) cells. Cell viability was not significantly affected at concentrations ≤50 μM Tempol. However, concentrations of 100 and 200 μM Tempol decreased cell viability significantly compared to the viability of the control cells (filled circles). HEI-OC1 cells were pretreated with different concentrations of Tempol for 2 h and then exposed to 30 μM cisplatin for 24 h (empty circles). HEI-OC1 cells were maximally protected by treatment with 50 μM Tempol. * Optimal experimental concentration of Tempol (**A**). HEI-OC1 cells were pretreated with 50 μM Tempol for 2 h and then exposed to 30 μM cisplatin for 24 h. Tempol exerted a significant protective effect against cisplatin cytotoxicity (**B**, * *p* < 0.05; paired Student’s *t*-test). Each group was tested in triplicate. CP, cisplatin; TP, Tempol.

To determine the effect of Tempol on cell viability, we used the 3-[4,5-dimethylthiazol-2-yl]-2,5-diphenyltetrazolium bromide (MTT) assay to first measure the viability of HEI-OC1 cells after 24-h exposure to different concentrations of Tempol. Exposure to 5, 10, 25, 50, 100, and 200 μM of Tempol resulted in survival rates of 104.8% ± 1.8%, 97.8% ± 4.9%, 97.3% ± 5.3%, 96.0% ± 5.2%, 90.3% ± 3.1%, and 86.2% ± 2.7%, respectively. There was no significant change in cell viability after exposure up to 50 μM Tempol. Exposure of cells to 30 μM cisplatin for 24 h after a 2-h pre-treatment with the same concentrations of Tempol resulted in survival rates of 65.6% ± 2.1%, 69.0% ± 1.9%, 82.0% ± 4.3%, 83.9% ± 3.9%, 79.7% ± 6.8%, and 75.3% ± 5.1%, respectively (Figure 1A). Pre-treatment with 50 μM Tempol showed the highest protective effect against cisplatin toxicity; therefore, this concentration was used for the subsequent experiments.

### 2.2. Protective Effect of Tempol against Cisplatin Cytotoxicity

HEI-OC1 cells were divided into the following treatment groups: control, 30 μM cisplatin alone, 50 μM Tempol alone, and 30 μM cisplatin after 50 μM Tempol pre-treatment for 2 h. After the cells were cultured for 24 h, cell viability was determined using the MTT assay. The cell viabilities in the cisplatin-alone, Tempol-alone, and cisplatin exposure after Tempol pretreatment groups were 59.2% ± 3.1%, 96.0% ± 5.2%, and 83.9% ± 3.9%, compared to cell viability in the control group. Cell viability in the cisplatin exposure after Tempol pre-treatment group was significantly higher than that in the cisplatin-alone group (*p* < 0.05, Figure 1B).

### 2.3. Changes in Apoptotic Characteristics of Cells

**Figure 2:**
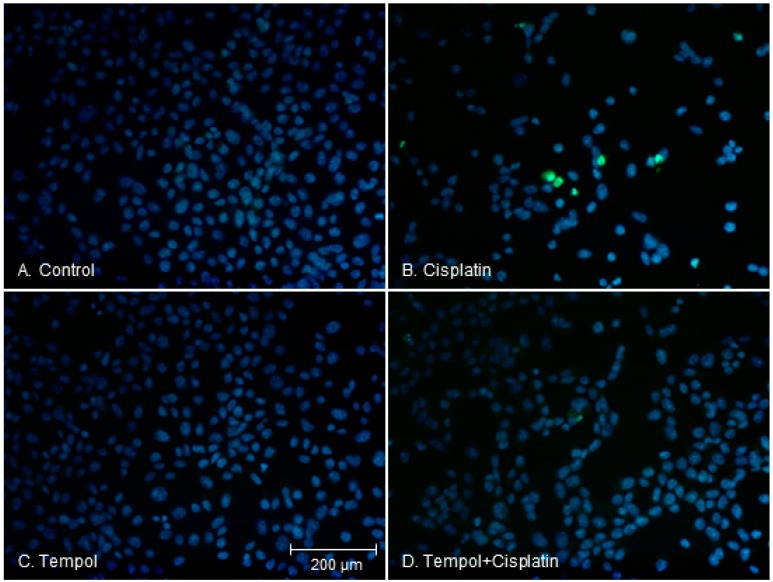
Apoptotic features in HEI-OC1 cells were decreased by pre-treatment with 50 μM Tempol for 2 h before cisplatin exposure for 24 h. The control (**A**) and Tempol-treated cells (**C**) show round-shaped nuclei with homogeneous intensity; Cisplatin-exposed cells show fragmented nuclei and apoptotic properties (green fluorescence) (**B**); Fewer cells display these features in the group pretreated with Tempol prior to cisplatin exposure (**D**). Four figures have the same scale bars.

To compare the morphological characteristics of apoptotic cell death, we divided HEI-OC1 cells into a control group, cisplatin-alone group, Tempol-alone group, and cisplatin exposure after Tempol pre-treatment group. After 24 h, the morphological characteristics of the cells were compared using the terminal deoxynucleotidyl transferase dUTP nick end labeling of nuclei (TUNEL) assay. The cells in the control and Tempol-alone groups had round nuclei with homogeneous intensity. Cells in the cisplatin-alone group showed fragmented nuclei and apoptotic properties (localized green fluorescence within the nucleus of cells), whereas fewer cells displayed these properties in the cisplatin exposure after Tempol pre-treatment group (Figure 2).

### 2.4. Changes in the Number of Apoptotic Cells

**Figure 3:**
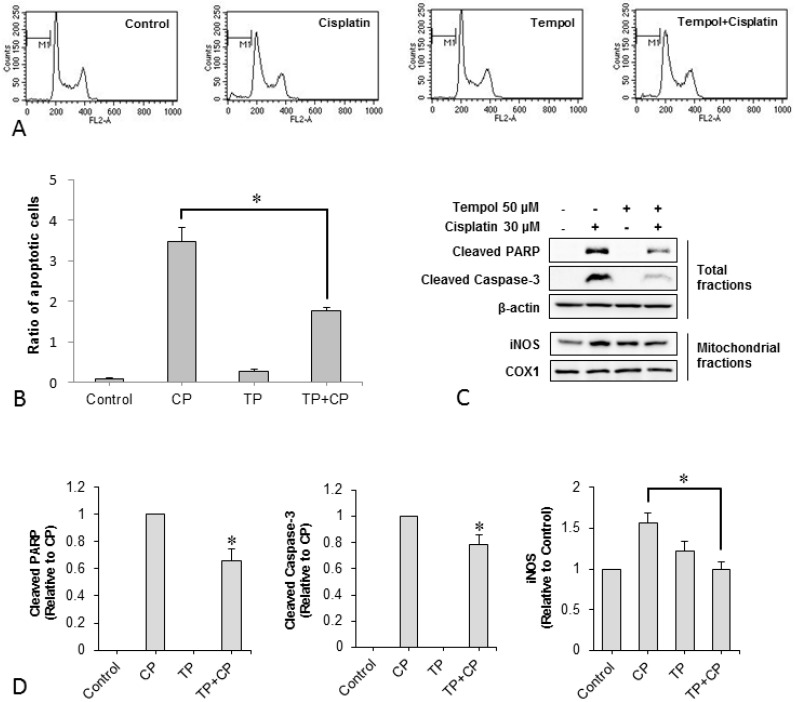
The ratio of apoptotic cells was determined using flow cytometry and representative histograms are illustrated (**A**). Apoptotic cells were reduced by 2-h pre-treatment with 50 μM Tempol before cisplatin exposure for 24 h (**B**, * *p* < 0.05 compared to cells treated with cisplatin alone, paired Student’s *t*-test). Expression of cleaved PARP, cleaved caspase-3, and iNOS increased in the cisplatin-treated group. However, Tempol inhibited the cisplatin-induced increase in expression of cleaved PARP, cleaved caspase-3, and iNOS. (**C**,**D**, * *p* < 0.05, paired Student’s *t*-test). Each group was tested in triplicate. COX1, cyclooxygenase 1; CP, cisplatin; FL2-A, fluorescence channel 2 area; iNOS, inducible nitric oxide synthase; PARP, poly ADP-ribose polymerase; TP, Tempol.

In order to determine whether apoptosis, the main form of cell death caused by cisplatin cytotoxicity, is suppressed by pre-treatment with Tempol, the ratio of apoptotic to non-apoptotic cells was determined using flow cytometry. Representative histograms are shown in Figure 3A. The ratios of apoptotic to non-apoptotic cells were 0.1 ± 0.1 in the control group, 3.5 ± 0.3 in the cisplatin-alone group, 0.3 ± 0.1 in the Tempol-alone group, and 1.8 ± 0.2 in the cisplatin exposure after Tempol pre-treatment group. Thus, cisplatin increased the ratio of apoptotic to non-apoptotic cells, whereas Tempol pre-treatment significantly reduced this ratio (*p* < 0.05, Figure 3B).

### 2.5. Changes in Expression of the Apoptotic Proteins Cleaved Poly ADP-Ribose Polymerase (PARP), Cleaved-Caspase 3, and Mitochondrial Inducible Nitric Oxide Synthase (iNOS)

In order to examine whether Tempol pre-treatment suppresses cisplatin-induced apoptotic changes, the protein levels of cleaved PARP and cleaved caspase-3, which are the active forms of representative apoptotic markers were investigated. Cisplatin induced the expression of cleaved PARP and cleaved caspase-3 proteins, whereas Tempol pre-treatment significantly inhibited this cisplatin-induced increase in expression. In addition, iNOS expression in isolated mitochondrial proteins was measured to determine the effect on cisplatin-induced oxidative stress on mitochondria. Cisplatin elevated mitochondrial iNOS expression, whereas Tempol pre-treatment significantly inhibited this cisplatin-induced increase in expression (Figure 3C,D).

### 2.6. Changes in Intracellular ROS Levels

**Figure 4:**
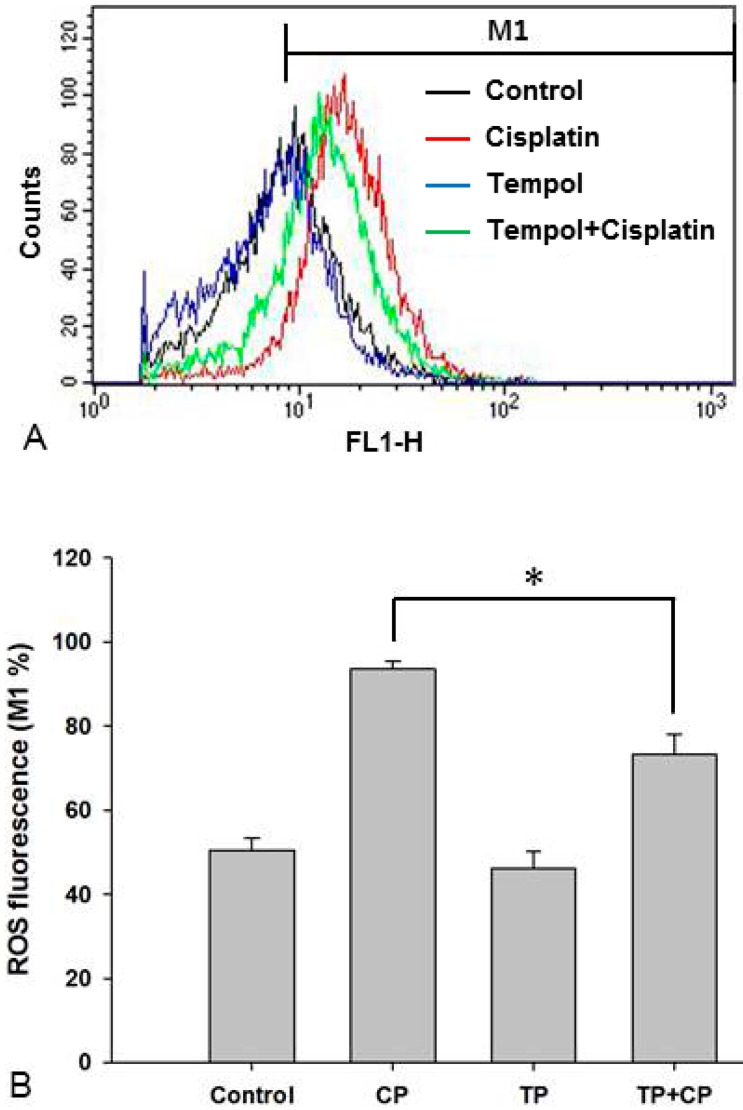
Intracellular reactive oxygen species (ROS) levels were determined by flow cytometry and representative histograms of ROS fluorescence are illustrated in (**A**). ROS production increased in the cisplatin-alone group. However, this increase in ROS levels was significantly inhibited in the cisplatin exposure after Tempol-pretreatment group (**B**, * *p* < 0.05, paired Student’s *t*-test). Each group was tested in triplicate. CP, cisplatin; FL1-H, fluorescence channel 1 height; M1, marker 1; TP, Tempol.

In order to determine whether Tempol pre-treatment suppresses cisplatin-induced increases in ROS levels, the levels of intracellular ROS in the four groups were measured. ROS levels in the control group, cisplatin-alone group, Tempol-alone group, and cisplatin exposure after Tempol pre-treatment group were 50.5% ± 2.9%, 93.7% ± 1.7%, 46.2% ± 4.0%, and 73.2% ± 4.9%, respectively. The cisplatin-induced increase in ROS levels was significantly decreased by pre-treatment with Tempol before cisplatin exposure (*p* < 0.05, Figure 4).

### 2.7. Mitochondrial and Nuclear Changes

**Figure 5:**
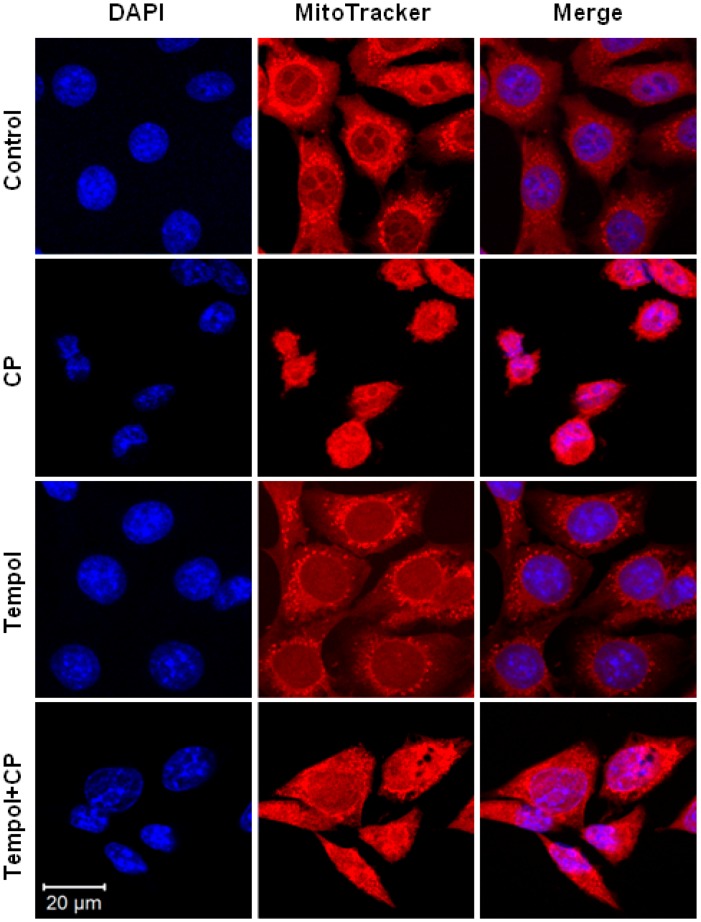
Confocal microscopic evaluation of the mitochondria and nuclei in HEI-OC1 cells. Cells seeded on coverslips were treated with 30 μM cisplatin for 24 h with or without pretreatment with 50 μM Tempol for 2 h. Controls cells were not exposed to cisplatin or Tempol. The mitochondrial structures were detected by MitoTracker^®^ staining (red) in the control and Tempol-alone groups. In contrast, the mitochondrial structure decreased in the cisplatin-alone group (CP). Pre-treatment with Tempol before cisplatin exposure inhibited the cisplatin-induced mitochondrial and nuclear changes (Tempol + CP). All figures have the same scale bars.

In order to investigate whether Tempol pre-treatment suppresses cisplatin-induced mitochondrial changes, the mitochondrial structures in the cells of the different treatment groups were compared using fluorescent staining. In the control group, 4′,6-diamidino-2-phenylindole (DAPI)-stained nuclei and mitochondrial networks, which were stained red, were observed in the cytoplasm. Similarly, cells in the Tempol-alone group also showed well-maintained mitochondrial networks. In contrast, cells in the cisplatin-alone group showed altered nuclear shapes as well as a reduction in the mitochondrial network in the cytoplasm. Mitochondrial networks were maintained in the cisplatin exposure after Tempol pre-treatment group when compared to those in the cisplatin-alone group (Figure 5).

### 2.8. Changes in the Mitochondrial Membrane Potential

**Figure 6:**
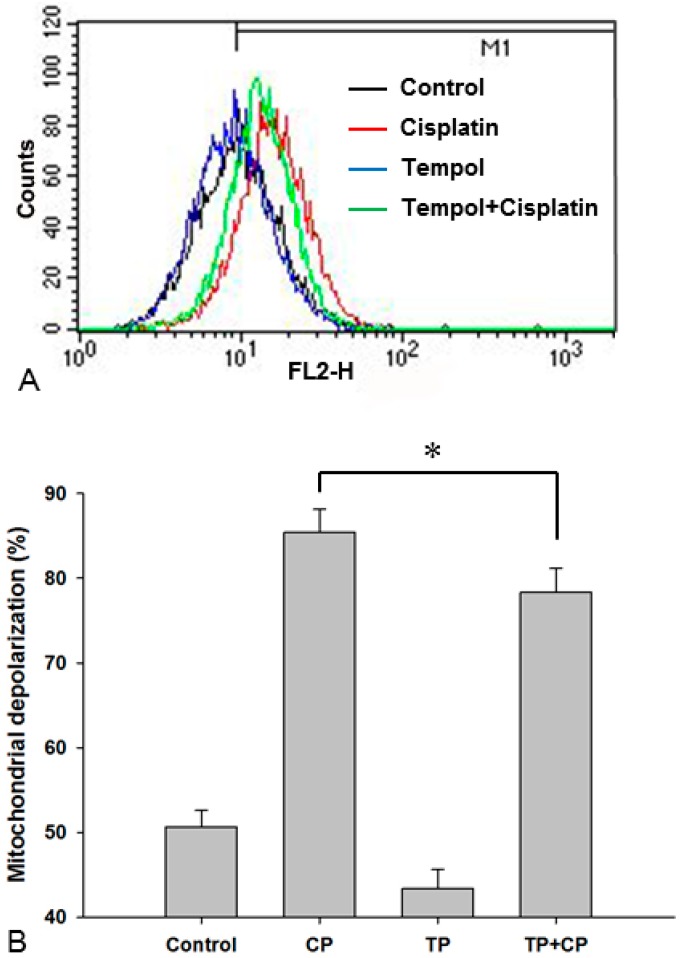
Mitochondrial membrane potential was determined by flow cytometry and representative histograms of mitochondrial membrane potential intensity are illustrated in (**A**). Exposure to 30 μM cisplatin alone for 24 h increased mitochondrial depolarization. This increase in mitochondrial depolarization was significantly inhibited in cells pretreated with 50 μM Tempol for 2 h before cisplatin exposure (**B**, *p < 0.05, paired Student’s *t*-test). Each group was tested in triplicate. CP, cisplatin; FL2-H, fluorescence channel 2 height; TP, Tempol.

In order to identify whether Tempol pre-treatment suppresses cisplatin-induced mitochondrial changes, the mitochondrial membrane potential (%) was measured. The mitochondrial membrane potential values were 50.7% ± 1.9% in the control group, 85.4% ± 2.7% in the cisplatin-alone group, 43.4% ± 2.2% in the Tempol-alone group, and 78.3% ± 2.8% in the cisplatin exposure after Tempol pre-treatment group. These results indicated that mitochondrial depolarization was increased significantly by cisplatin and that this increase was significantly inhibited by Tempol pre-treatment (*p* < 0.05, Figure 6).

## 3. Discussion

While cisplatin is a representative anticancer drug that is widely used for treatment of solid tumors, its use is limited by the fact that it can cause permanent and irreversible hearing loss. Several mechanisms are involved in cisplatin-induced ototoxicity. The most well-known mechanism is the induction of ROS production, which causes oxidative stress and lipid peroxidation, resulting in apoptosis of hair cells, supporting cells, stria vascularis, and auditory nerves [3,8]. Once ROS production is induced, endogenous antioxidants in the cochlea, including glutathione, superoxide dismutase (SOD), catalase, and glutathione peroxidase, are activated to counteract the increase in ROS levels [9]. However, continuous exposure to cisplatin results in excessive accumulation of ROS, which inhibits synthesis of endogenous antioxidants. Tempol, a stable nitroxide compound, is a transmembranous radical scavenger that functions in a manner similar to SOD and interacts with superoxide anions to generate hydrogen peroxides [10]. Since Tempol has a low molecular weight (MW: 172), it easily penetrates cellular membranes to scavenge intracellular superoxide anions [11,12]. Moreover, because it can penetrate the blood–brain barrier more easily than other antioxidants, Tempol can exert a neuroprotective effect [7]. To date, there have been several reports on the various protective effects of Tempol against inflammation or oxidative damage in tissues [12,13]. In particular, Tempol exerts neuroprotective effects in models of traumatic brain injury, ischemic stroke, and Parkinson’s disease because of its ability to penetrate the blood–brain barrier more easily than other antioxidants can [14,15,16].

The anti-oxidative effect of Tempol is comparable to that of *N*-acetyl cysteine (NAC), an antioxidant that is well known for its scavenging activity on cellular superoxide anions. However, NAC increases the levels of superoxide anions at low concentrations whereas Tempol does not, making Tempol more effective for the protection of cells from oxidative stress [10,17]. In addition, Tempol, like catalase, promotes hydrogen peroxide metabolism and suppresses the generation of toxic hydroxyl radicals [18]. Superoxide anions and nitric oxide (NO) produce ONOO−, a strong oxidant that causes cellular damage, which can be inhibited by Tempol [19]. In order to investigate the protective effect of Tempol against cisplatin-induced cytotoxicity, we measured intracellular ROS levels in the present study and found that Tempol pre-treatment significantly inhibited the cisplatin-induced increase in ROS levels.

Superoxide anions aid in pro-apoptotic Bax protein movement into the cytoplasm. This results in cytochrome c liberation due to mitochondrial wall damage and activation of caspase-3, which in turn, results in apoptosis [9]. In addition, ROS induces the expression of transient receptor potential vanilloid ion channels, which increases calcium inflow into cells, leading to the activation of caspase-3 [20]. The present study also showed that the cisplatin-induced expression of two apoptotic proteins, cleaved caspase-3 and cleaved PARP, was suppressed by Tempol pre-treatment, thereby demonstrating its anti-apoptotic effect. Movement of pro-apoptotic Bax protein into the cytoplasm aided by superoxide anions leads to the formation of pores on the mitochondrial membrane, which results in a decrease in the mitochondrial transmembrane potential [21] and an increase in mitochondrial depolarization [22,23]. In the present study, the cisplatin-induced increase in mitochondrial damage and mitochondrial depolarization was reduced by Tempol pre-treatment, thereby indicating that Tempol also exerts an inhibitory effect against mitochondrial damage caused by cisplatin-induced oxidative stress.

Other mechanisms of cisplatin-induced cytotoxicity include cisplatin binding to the guanine base of DNA, which induces p53 activation and delays cell cycle progression, thereby causing apoptosis [24]. In the present study, when we investigated the ratio of apoptotic to non-apoptotic cells through cell cycle analysis using flow cytometry, we found that cisplatin treatment increased the ratio of apoptotic cells. This cisplatin-induced increase in apoptosis was suppressed by Tempol pre-treatment, thereby demonstrating the protective effect of Tempol against cisplatin-induced cytotoxicity.

Minami et al. [25] found that administration of Tempol to noise-exposed guinea pigs improved the hearing threshold and inhibited the accumulation of free radicals involved in noise-induced cell death, thereby exerting a protective effect against noise-induced hearing loss. Furthermore, orally administered Tempol reached the cochlea by crossing the blood–brain barrier. Murashita et al. [26] reported that Tempol prevented noise-induced cochlear damage in mice. Nagashima et al. [27] reported that noise caused accumulation of ROS and NO, leading to damage of the spiral ligament. The mechanism underlying this effect was up-regulation of the c-Jun N-terminal kinase pathway and down-regulation of connexin 26, which was inhibited by Tempol administration, thereby preventing hearing loss. The effect of co-administration of Tempol with cisplatin on the suppression of tumor growth in mice was similar to that observed in mice treated with cisplatin alone. Therefore, Tempol did not interfere with the anti-cancer effect of cisplatin against the growth of solid carcinomas [4].

The present study demonstrated that Tempol effectively suppressed cell damage and oxidative stress caused by cisplatin, an ototoxic drug. To date, many antioxidants, such as ROS scavengers and anti-inflammatory drugs have been studied for use as protective drugs against cisplatin-induced ototoxicity [28]; however, no clinical trials have been conducted. Tempol has a relatively high blood–brain barrier penetrating ability because its molecular weight is lower than that of existing agents. Therefore, it can potentially be used as a protective agent against cisplatin-induced ototoxicity.

## 4. Materials and Methods

### 4.1. Cell Culture

The House Ear Institute-Organ of Corti 1 (HEI-OC1) cell line is extremely sensitive to ototoxic drugs and expresses specific markers that are characteristic of organ of Corti cells. Therefore, this cell line has been used as an in vitro system to investigate the mechanisms underlying ototoxicity and to screen new drugs for otoprotective properties [29]. HEI-OC1 cells were cultured in high-glucose Dulbecco’s modified Eagle’s medium (GIBCO BRL, Grand Island, NY, USA) with 10% fetal bovine serum (Lonza, Walkersville, MD, USA). Cells were allowed to continuously proliferate without differentiation in a 5% CO_2_ environment at 33 °C. The cells were exposed to cisplatin or Tempol (Sigma, St. Louis, MO, USA) for 24 h in culture, and the Tempol pre-treatment group was pre-treated with Tempol for 2 h and then exposed to cisplatin for 24 h in culture.

### 4.2. MTT Assay

The MTT (3-[4,5-dimethylthiazol-2-yl]-2,5-diphenyltetrazolium bromide) assay was performed to measure cell viability. HEI-OC1 cells were cultured in a 24-well plate at a density of 3 × 10^4^ cells per well. The cultured cells were treated with Tempol at 5, 10, 25, 50, 100, and 200 μM for 24 h or pretreated with each concentration of Tempol for 2 h, and then exposed to 30 μM cisplatin for 24 h. Then, 450 µL of the cell suspension from each well was treated with 50 μL of 5 mg/mL MTT solution (Sigma, St. Louis, MO, USA) and incubated under 5% CO_2_ at 33 °C for another 4 h. After centrifugation of the formazan crystals, 500 µL dimethyl sulfoxide was added to each well, and after 15 min, absorbance at 570 nm was measured using a spectrophotometer (BioTek, Winooski, VT, USA). Three independent sets of experiments were performed with each treatment. The cell viability for each treatment group was calculated as a percentage of the viability of the control cells by using the following formula: optical density in the test well × 100/optical density in the control well.

### 4.3. TUNEL Assay

The terminal deoxynucleotidyl transferase dUTP nick end labeling of nuclei (TUNEL) assay was performed to detect DNA fragmentation in apoptotic cells. HEI-OC1 cells were cultured in a 6-well plate with three slides at a density of 3 × 10^5^ cells per well. The cells were treated as follows: 30 µM cisplatin alone for 24 h, 50 µM Tempol alone for 24 h, or pre-treatment with 50 µM Tempol for 2 h followed by exposed to 30 µM cisplatin for 24 h. The assay was performed using the DeadEnd^TM^ Fluorometric TUNEL System (Promega, Madison, WI, USA) according to the manufacturer’s protocol. Images were taken using a fluorescence microscope (Olympus IX71, Tokyo, Japan).

### 4.4. Apoptosis Analysis Using Flow Cytometry

Cells that adhered to the bottom of the culture vessel were collected after trypsin treatment and centrifuged at 2000 rpm for 3 min. Following removal of the supernatant medium, the cells were then washed in ice-cold phosphate-buffered saline (PBS) and fixed in 70% cold ethanol at 4 °C for 30 min. After washing twice with PBS, the cells were incubated with propidium iodide (PI) solution (50 µg/mL PI, 50 µg/mL RNase A, and 0.05% Triton X-100 in PBS) for 15 min. Fluorescence intensities of the DNA in PI-stained cells were analyzed using flow cytometry (FACSCalibur, BD Biosciences, San Diego, CA, USA), and the amount of DNA was calculated on the basis of the fluorescence intensity by using Cell Quest software (BD Biosciences). Each group was tested in triplicate. The cells with the fluorescence intensity lower than that of cells in the G0/G1 phase of the cell cycle were named Sub-G0/G1 cells and analyzed as apoptotic cells.

### 4.5. Western Blotting

After the indicated treatments, the cells were centrifuged at 2000 rpm for 3 min and the medium was removed. The cells were washed with PBS, incubated in lysis buffer (20 mM 4-(2-hydroxyethyl)-1-piperazine ethane sulfonic acid (HEPES); pH 7.4, 2 mM ethylene glycol tetra-acetic acid (EGTA), 50 mM glycerol phosphate, 1% Triton X-100, 10% glycerol, 1 mM dithiothreitol, 1 mM phenylmethylsulfonyl fluoride, 10 µg/mL leupeptin, 10 µg/mL aprotinin, 1 mM Na_3_VO_4_, and 5 mM NaF) at 0 °C for 10 min, and then centrifuged at 15,000 rpm to extract cellular proteins. The extracted proteins were quantified using a Bio-Rad dye-binding microassay (Bio-Rad, Hercules, CA, USA), heated with Laemmli sample buffer at 98 °C for 5 min, and then subjected to sodium dodecyl sulfate-polyacrylamide gel electrophoresis. The proteins were then transferred onto nitrocellulose membranes (Millipore, Darmstadt, Germany), and the membranes were incubated with TBST (20 mM Tris-HCl, pH 7.5, 137 mM NaCl, and 0.1% Tween-20) supplemented with 5% skim milk at room temperature for 1 h to block all non-specific binding. Subsequently, the membranes were incubated with primary antibodies. The primary antibodies used were specific to cleaved poly ADP-ribose polymerase (PARP), cleaved caspase-3, inducible nitric oxide synthase (iNOS; Cell Signaling Technology, Danvers, MA, USA), Cyclooxygenase 1 (COX1), and β-actin (Santa Cruz Biotechnology, Santa Cruz, CA, USA). The membranes were incubated overnight at 4 °C with each antibody diluted at a ratio of 1:1000 in TBST buffer. After washing four times with TBST for 15 min, the membranes were incubated with secondary antibody diluted at a ratio of 1:4000 in 5% skim milk solution for 2 h, followed by washing four times with TBST. The secondary antibodies used were anti-mouse antibodies produced in sheep, anti-rabbit antibodies produced in donkeys (Jackson Immunoresearch, West Grove, PA, USA), and anti-goat antibodies produced in donkeys (Santa Cruz Biotechnology). Protein bands were detected using a western blot detection system (iNtRON Biotechnology, Daejun, Korea), and images were evaluated using an Image analyzer (LAS-3000 Imaging System, FujiFilm, Tokyo, Japan). Relative protein band quantification was performed using mean optical density of the bands. Data are presented as a ratio of target protein to β-actin or COX1, which were used as loading controls. All experiments were repeated three times to confirm the results.

### 4.6. Intracellular ROS Measurement

HEI-OC1 cells were cultured in a 6-well plate at a density of 3 × 10^5^ cells per well in an incubator with 5% CO_2_ at 33 °C. The cells in the culture vessel were collected by trypsin treatment and centrifuged at 1000 rpm for 3 min, followed by removal of the supernatant and washing with PBS. The cells were then resuspended in a 10 μM CM-H_2_DCFDA (fluorochrome marker 5-(and-6)-chloromethyl-2′,7′-dichlorodihydro-fluorescein diaceate, acetyl ester; Invitrogen Molecular Probes, Eugene, OR, USA) solution and incubated under 5% CO_2_ at 33 °C for 15 min. After washing twice with PBS, the fluorescence intensity of the cells was measured using flow cytometry (FACSCalibur, BD Biosciences). ROS levels were analyzed with Cell Quest software (BD Biosciences), and the mean values of the measured fluorescence intensities were used for comparative analysis. Each group was tested in triplicate.

### 4.7. Confocal Microscopic Evaluation of Mitochondria

HEI-OC1 cells were cultured in a 6-well plate containing poly-l-lysine (Sigma, St. Louis, MO, USA)-coated 12-mm glass coverslips (Deckglaser, Braunschweig, Germany). Following cisplatin and Tempol treatments, the cells were cultured in an incubator with 5% CO2 at 33 °C for 24 h. The cells were stained with 200 nM MitoTracker^®^ Red CMXRos (Invitrogen, Waltham, MA, USA) in serum-free medium for 20 min. The stained cells were washed with PBS and fixed using 1 mL of 3.7% formaldehyde at room temperature for 15 min. After washing the fixed cells twice with PBS, the glass coverslip with adhered cells was fixed on the slide using fluorescent mounting medium containing 4′,6-diamidino-2-phenylindole (DAPI; GBI Labs, Mukilteo, WA, USA). Images were acquired using a Zeiss LSM 510 Meta confocal microscope (Carl Zeiss, Oberkochen, Germany).

### 4.8. Analysis of the Mitochondrial Membrane Potential

The mitochondrial membrane potential was measured using MitoTracker^®^ Red CMXRos (Invitrogen). HEI-OC1 cells were treated with cisplatin and Tempol and then cultured for 24 h, followed by staining with 200 nM MitoTracker^®^ Red CMXRos (Invitrogen) in serum-free medium for 20 min. The cells were detached by trypsin treatment and centrifuged at 1000 rpm for 3 min to remove the medium. After washing twice with PBS, the fluorescence intensity of the cells was measured using flow cytometry (FACSCalibur, BD Biosciences) and analyzed using Cell Quest software (BD Biosciences). Each group was tested in triplicate.

### 4.9. Isolation of the Mitochondria

Cultured cells were centrifuged at 2000 rpm for 3 min to remove the medium. The cells were then washed with ice-cold PBS and incubated with 1 mL cytoplasm extraction buffer (1 mM HEPES at pH 7.9, 1.5 mM MgCl_2_, 0.2 mM dithiothreitol, 1 mM phenylmethylsulfonyl fluoride, 10 μg/mL leupeptin, 10 μg/mL aprotinin, 1 mM Na_3_VO_4_, and 5 mM NaF, 0.2% NP-40) on ice for 5 min. After incubation, the cells were passed through a 1-mL syringe with a 25-gauge needle 20 times. Cell debris was removed by centrifugation at 13,000 rpm for 15 min at 4 °C. The supernatant was again subjected to centrifugation at 80,000 rpm for 15 min, and the pelleted mitochondria were washed with 1 mL cytoplasm extraction buffer to remove proteins. Subsequently, mitochondria were resuspended in 100 μL lysis buffer (20 mM HEPES; pH 7.4, 2 mM EGTA, 50 mM glycerol phosphate, 1% Triton X-100, 10% glycerol, 1% NP-40, 1 mM dithiothreitol, 1 mM phenylmethylsulfonyl fluoride, 10 µg/mL leupeptin, 10 µg/mL aprotinin, 1 mM Na_3_VO_4_, and 5 mM NaF). Mitochondria were sonicated twice for 3 s to increase the purity of the fraction, and then centrifuged at 13,000 rpm for 15 min at 4 °C to separate mitochondrial proteins in the supernatant.

### 4.10. Statistical Analysis

The results were statistically analyzed using SPSS 19.0 software (SPSS Inc., Chicago, IL, USA). Student’s paired *t*-test was used to analyze the data. A *p* value <0.05 was considered statistically significant. Data are presented as mean ± standard deviation.

## 5. Conclusions

In conclusion, in the present study, we found that Tempol pre-treatment had a protective effect against cell damage due to cisplatin-induced oxidative stress; therefore, Tempol can be used for the development of protective agents against cisplatin-induced ototoxicity.

## Abbreviations

HEI-OC1	House Ear Institute-Organ of Corti 1
MTT	3-[4,5-Dimethylthiazol-2-yl]-2,5-diphenyltetrazolium bromide
PARP	Poly ADP-ribose polymerase
NOS	Nitric oxide synthase
ROS	Reactive oxygen species
SOD	Superoxide dismutase
CP	Cisplatin
TP	Tempol
NO	Nitric oxide
DMSO	Dimethyl sulfoxide
PBS	Phosphate buffered saline
PI	Propidium iodide
